# Obituary

**Published:** 1890-11

**Authors:** 


### Obituary.

DR. WILLIAM VAN PELT, of Williamsville, died at his home on Sunday, October 12, 1890, aged seventy-five years. He had, since 1839, engaged in the practise of his profession in this county, and was, at the time of his death, the oldest living member of the Erie County Medical Society, of which he had been president. Dr. Van Pelt was a brother-in-law of Dr. E. Storck, of this city, and leaves seven children, two of whom are physicians, viz., Dr. J. G. Van Pelt of Suspension Bridge, N. Y., and Dr. C. L. Van Pelt, of Toledo, Ohio. The funeral was largely attended by relatives, friends and acquaintances of the deceased, from the family residence on Wednesday, October 15, 1890.

